# Complete Genome Sequence of K14JB01, a Novel Variant Strain of Porcine Epidemic Diarrhea Virus in South Korea

**DOI:** 10.1128/genomeA.00505-14

**Published:** 2014-05-29

**Authors:** Yoon-Young Cho, Seong-In Lim, Yong Kwan Kim, Jae-Young Song, Joong-Bok Lee, Dong-Jun An

**Affiliations:** a Animal and Plant Quarantine Agency, Anyang, Gyeonggi-do, Republic of Korea; b Department of Infectious Diseases, College of Veterinary Medicine, Konkuk University, Seoul, Republic of Korea

## Abstract

A novel variant strain of porcine epidemic diarrhea virus (PEDV) emerged on pig farms in South Korea during late 2013. Genomic DNA isolated from a K14JB01 strain identified in a diarrheal pig showed high sequence similarity to PEDV strains prevailing in the United States in 2013. This is the first study to identify the complete genome sequence of a novel variant PEDV in South Korea.

## GENOME ANNOUNCEMENT

Porcine epidemic diarrhea virus (PEDV) belongs to the family *Coronaviridae*; it is an enveloped, single-stranded RNA virus that causes enteritis, vomiting, and watery diarrhea (1). PEDV was first detected in Belgium and the United Kingdom in 1978 (2, 3), and has since been identified in Hungary, Italy, China, and South Korea (4–7). In 2013, PEDV spread throughout the pig population in the United States, and the viral genome showed a close relationship with that of variant strains identified in China (8). Frequent outbreaks of PED have occurred in South Korea since the early 1990s; however, piglets infected with PEDV from November 2013 suffered from several enteric diseases, resulting in a high mortality. Here, the complete sequence of a novel variant PEDV strain, K14JB01 (isolated from an infected pig in South Korea), was sequenced and analyzed.

In January 2014, fecal samples were collected from pigs with diarrhea in Gyeonggi Province, South Korea, and viral RNA was extracted using the RNeasy Minikit (cat no. 74104; Qiagen) according to the manufacturer’s instructions. cDNA was prepared using an OneStep reverse transcription (RT)-PCR kit (cat no. 210210; Qiagen). Twenty sets of primers were designed (based on the USA/Iowa/18984/2013 strain) to cover the full-length PEDV genome. The amplified PCR products were cloned into the pGEM-T Easy Vector (Promega, USA) and then sequenced at the Cosmogentech Institute using an ABI Prism 3730xi DNA sequencer. All fragments were then assembled and edited using Clustal X 1.83 (9) to yield the complete genome sequence.

The genome of PEDV strain K14JB01 is 28,038 nucleotides (nt) in length, excluding the 3′ poly (A) tail, and is arranged as follows: a 5′ untranslated region (UTR) comprising 292 nt, an open reading frame 1a (ORF1a) and ORF1b encoding a replicase of 20,345 nt, a spike (S) gene comprising 4,161 nt, an ORF 3 comprising 675 nt, an envelope (E) gene comprising 231 nt, a membrane (M) gene comprising 681 nt, a nucleocapsid (N) gene comprising 1,326 nt, and a 3′ UTR comprising 333 nt.

The complete genome of PEDV strain K14JB01 showed high nucleotide sequence homology (99.7 to 99.8%) with variant U.S. strains (USA/Colorado/2013, USA/Iowa/18984/2013, and USA/Indiana/17846/2013, IA1, IA2, and MN) identified in 2013, and with Chinese strains (AH2012, BJ-2011-1, GD-B, and JS-HZ2012) identified in 2011 and 2012 (99.1 to 99.4%). However, K14JB01 showed relatively low sequence homology (96.3 to 97.6%) with Korean vaccine strains (SM98 and DR13) and the CV777 strain.

The ORF1a and ORF1b genes of K14JB01 show 99.8% homology (at the nucleotide level) with those of USA/Iowa/16465/2013; in addition, the S genes are 99.6% similar, the ORF3 genes are 100% identical, the M genes are 99.9% similar, and the N genes are 99.9% similar.

In summary, PEDV strain K14JB01 identified from a diarrheal pig in South Korea shows high nucleotide sequence homology with variant strains circulating in the United States and China. The complete genome sequence data of K14JB01 will be the basis of our understanding of the genomic characterization and will contribute to the elucidation of PEDV in South Korea.

### Nucleotide sequence accession number.

The complete genome sequence of PEDV strain K14JB01 was deposited in GenBank under accession no. KJ623926.
